# Proteomic identification of differentially expressed and phosphorylated proteins in epidermis involved in larval-pupal metamorphosis of *Helicoverpa armigera*

**DOI:** 10.1186/1471-2164-10-600

**Published:** 2009-12-12

**Authors:** Qiang Fu, Peng-Cheng Liu, Jin-Xing Wang, Qi-Sheng Song, Xiao-Fan Zhao

**Affiliations:** 1 School of Life Sciences, the Key Laboratory of Plant Cell Engineering and Germplasm Innovation, Ministry of Education, Shandong University, Jinan, 250100, PR China; 2 Division of Plant Sciences, University of Missouri, Columbia, Missouri 65211, USA

## Abstract

**Background:**

Metamorphosis is an important process in the life cycle of holometabolous insects and is regulated by insect hormones. During metamorphosis, the epidermis goes through a significant transformation at the biochemical and molecular levels.

**Results:**

To identify proteins and phosphoproteins involved in this process, we separated and compared epidermal protein profiles between feeding larvae and metamorphically committed larvae using two-dimensional gel electrophoresis and Pro-Q Diamond Phosphoprotein Staining. Sixty-one spots showing differential expression and/or phosphorylation were analyzed by mass spectrometry and eighteen proteins were proved related to larval-pupal transformation. Eight of them were further examined at the mRNA level by Reverse Transcription Polymerase Chain Reaction (RT-PCR) and two of them were examined at the protein level by Western blot. Calponin was highly expressed in the metamorphic epidermis and phosphorylated by protein kinase C.

**Conclusion:**

Our results suggest that the expression and phosphorylation of these proteins may play important roles in coordinating the biochemical processes involved in larval-pupal metamorphosis.

## Background

Metamorphosis, the complete transformation from larva to pupa and then to adult, is a significant process for holometabolous insects such as moths and flies. During metamorphosis larval tissues like midgut, fat body and integument are histolyzed via a programmed cell death process and remodeled to adult structures [1]. Insect metamorphosis is known to be controlled by the interplay of two hormones, juvenile hormone (JH) and 20-hydroxyecdysone (20E). At the end of the final larval stage, the JH titer decreases and 20E initiates metamorphosis in the absence of JH [2]. Recently, a set of transcriptional regulators involved in the ecdysone-regulated metamorphosis cascade has been revealed. For example, broad gene (Br) plays a key role in the initiation and progression through metamorphosis, but not in larval molt [1]. During this process, the phosphorylation state of these proteins is also closely related to their function. For instance, phosphorylation mediated by protein kinase C (PKC) can suppress JH action by preventing nuclear proteins from binding to JH-responsive promoters [3]. Moreover, PKC-mediated phosphorylation of ultraspiracle protein (USP) is essential for 20E-induced gene expression in the salivary glands of *Drosophila melanogaster *[4]. However, the molecular mechanism of hormonal regulation of metamorphosis is still a mystery. Therefore, it is important to identify specifically expressed proteins and their phosphorylation states in order to understand the biochemistry of metamorphosis.

Two-dimensional gel electrophoresis (2-DE) in conjunction with mass spectrometry provides a useful tool for detection of differentially expressed proteins. Zhang and his colleagues identified twelve proteins from the skeletal muscles of the silkworm, *Bombyx mori *[5] with significantly different expression levels in larvae and pupae. In the salivary gland of *Drosophila*, 20E-induced expression of fourteen proteins required PKC activity [6]. Recently, the Pro-Q Diamond Phosphoprotein Stain (Molecular Probes, United States) was found to be a powerful tool for revealing differentially regulated phosphoproteins and has been widely used to detect phosphoproteins after separation on 2-DE gels [7-9]. Thus, 2DE-based proteomic and phosphoproteomic approaches were applied to understand the molecular mechanisms of larval metamorphosis.

The cotton bollworm,*Helicoverpa armigera*, is an important agricultural pest worldwide. It is also an ideal model for studying the molting and metamorphosis of Lepidopteran insects because of its relatively large body size, fast development, and easy cultivation in the laboratory. Pauchet et al. reported the midgut lumen proteome of *H. armigera *feeding larvae and identified not only digestive enzymes but also arginine kinase and pathogen recognition proteins [10]. To identify the molting- and metamorphosis-related proteins and genes, we have reported a set of genes that are differentially expressed during molting and metamorphosis [11]. Thirty differentially expressed proteins, including enzymes, regulators, protein hydrolases and receptors, have been detected in the epidermis, fat body and hemolymph of *H. armigera *during larval molting [12]. The purpose of this study is to further identify the differentially expressed and phosphorylated proteins in the epidermis of *H. armigera *during larval-pupal metamorphosis.

## Results

### Mapping differentially expressed and phosphorylated proteins

**Figure 1:**
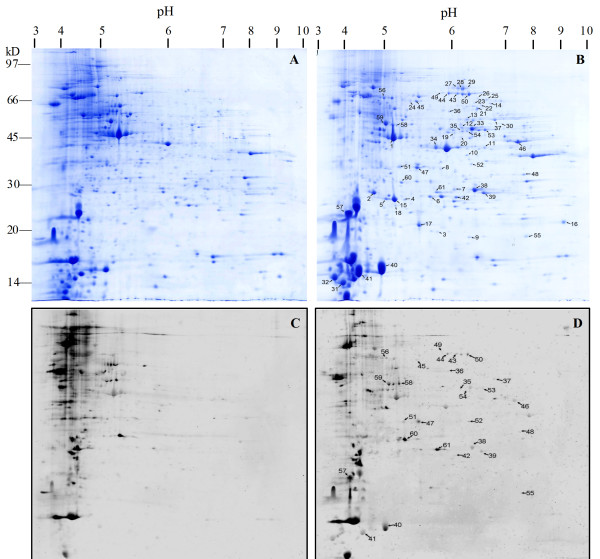
**Separation of proteins from *H. armigera *larval epidermis by 2-DE (2nd dimension: 12% SDS-PAGE)**. (A) 5th feeding (5th-F) larvae with Coomassie Brilliant Blue (CBB) stain. (B) 6th-metamorphically committed (6th-M) larvae with CBB staining. (C) 5th-F larvae with Pro-Q Diamond phosphoprotein gel (Pro-Q) stain. (D) 6th-M larvae with Pro-Q stain. Spots analyzed by mass spectrometry are designated by numbers. Spots annotated with the same number in both gels are the same spots visualized by different staining methods. The experiments were replicated three times.

Because the larva-pupa transition takes approximately 4 days, in order to identify as many metamorphically expressed proteins as possible, we combined the samples of metamorphosis (6th-M) epidermis from five time points during metamorphosis (see Materials and Methods). The 5th feeding larvae (5th-F) were selected as controls to compare the protein profiles between the metamorphosis and feeding samples. Instead of normalization of gels, the spots were compared by ratio (spot quantity/total spots quantity). Figure 1 showed the differentially expressed and phosphorylated proteins during metamorphosis.

**Figure 2:**
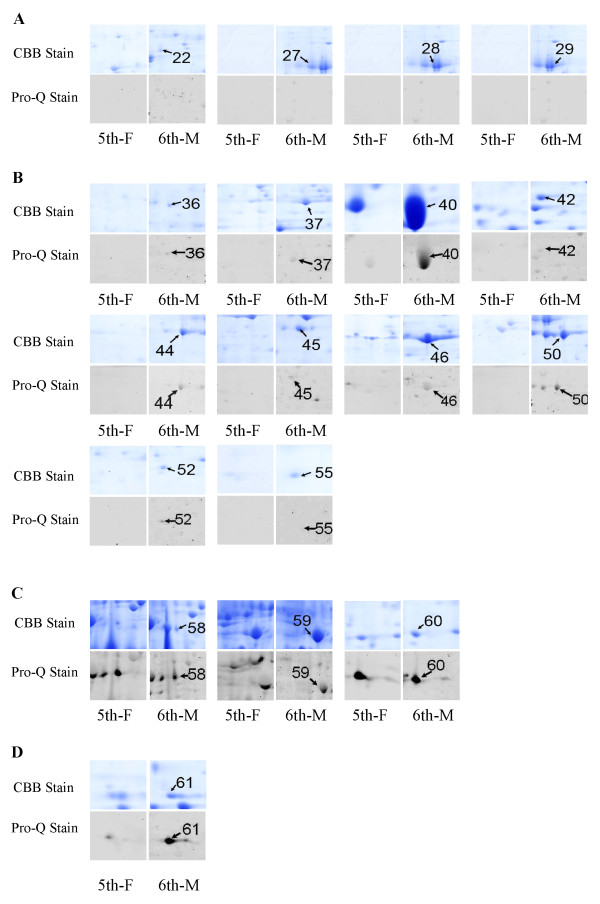
**Exhibition of spots from the 5th feeding larvae (5th-F) and 6th-metamorphically committed larvae (6th-M) stained with Coomassie Brilliant Blue (CBB stain) and Pro-Q diamond phosphoprotein stain (Pro-Q stain)**. (A) The protein expression was up-regulated but no phosphorylation was detected; (B) The protein expression was up-regulated and phosphorylation was also detected; (C) The protein expression remained the same but the phosphorylation decreased; (D) The protein expression remained the same but the phosphorylation increased.

The protein spots that were up-regulated on protein levels and/or had variation of phosphorylation in the 6th-M epidermis sample when compared with that of the 5th-F were selected for further identification by PMF (protein mass figerprint) and MS/MS spectra (Figure 1). These spots can be divided into three groups. The proteins in the first group had increased expression but were not phosphorylated because no signal was detected in the Pro-Q stained gels, including spots 1-34 (Figure 2A). In the second group, the increased expression of the proteins was accompanied by induced phosphorylation, including spots, 35-57 (Figure 2B). In the third group, the expression of the proteins was maintained at the same level, but their phosphorylation state changed, including spots 58-61, with a lower phosphorylation signal for spots 58, 59, and 60, but a higher signal for spot 61 in the 6th-M gel (Figure 2C, D).

### Identification of proteins by PMF and MS/MS spectra

In order to maximize protein identification, the NCBI nr_Metazoa Database was selected. Protein scores greater than 74 were considered statistically significant (p < 0.05) when using PMF spectra. In this way, sixteen spots were identified. When using MS/MS spectra, protein scores greater than 45 were statistically significant; five proteins were confirmed using this approach. In addition, 2 spots that were not identified by PMF data were identified using MS/MS spectra data. Because of the lack of information on *H. armigera *proteins, among the sixty-one spots analyzed by mass spectrometry only eighteen spots were identified by Mascot searches. Most of the proteins were identified according to protein sequences from other insects such as *D. melanogaster *and *B. mori*. Hexamerin (Spot 27, 28, 29) was the only one identified through *H. armigera *(Table 1). The other spots gave PMF and MS/MS spectra but Mascot searches did not produce significant match results.

**Table 1:** Proteins in the epidermis during metamorphosis identified by Mascot searches using protein mass fingerprints (PMF) and MS/MS against the NCBInr_Metazoa Database

		Description			PMF			MS/MS				
**Spot**	**Protein name**	**GenBank Accession number and organism**	**Theoretical MW(kD)/pI**	**Experimental MW(kD)/pI**	**Peptide hits**	Score*	**Coverage**	**Peptide hits**	Score*	**Peptide sequences**	**A**	**P**

22	viral-like chitinase	gi|74422655 [*Spodoptera exigua*]	61.6/6.14	64/6.55	27	211	38%	-	-	-	**↑ **	**-**
27	hexamerin	*gi*|50404096 [*Helicoverpa armigera*]	82.0/5.89	75/6.15	17	129	23%	-	-	-	**↑ **	**-**
28	hexamerin	Gi|50404096 [*Helicoverpa armigera*]	82.0/5.89	75/6.21	23	159	26%	-	-	-	**↑ **	**-**
29	hexamerin	Gi|50404096 [*Helicoverpa armigera*]	82.0/5.89	75/6.25	25	136	32%	-	-	-	**↑ **	**-**
36	prophenoloxidase-1	gi|159141810 [*Heliothis virescens*]	79.1/6.39	67/5.95	11	97	20%	-	-	-	**↑ **	**↑ **
37	arylphorin subunit	gi|5869989 [*Spodoptera litura*]	76.8/6.7	56/6.75	8	71	12%	2	55	RDPAFYQLYQRI KDLHQYSYEIIARH	**↑ **	**↑ **
40	heat shock protein 1	gi|56462154 [*Lonomia obliqua*]	21.9/6.32	16/4.95	9	111	38%	4	230	RNSYFRPWRT RLLDQHFGMGLKR RDWWDEWDRPSRL RHEEKQDEHGFISRQ	**↑ **	**↑ **
42	Phosphoglyceromu-tase	gi|1092224 [*Drosophila melanogaster*]	28.6/6.62	28/6.1	-	-	-	2	98	RILIAAHGNSLRG KYGEAQVQIWRR	**↑ **	**↑ **
44	aldehyde dehydrogenase	gi|164450344 *[Ectropis obliqua]*	22.0/5.67 (partial)	72/5.89	-	-	-	1	57	KFETFEEVVDRA	**↑ **	**↑ **
45	solute carrier family 25	gi|123232765 [*Mus musculus*]	55.0/8.81	67/5.55	12	77	24%	-	-	-	**↑ **	**↑ **
46	fructose 1,6-bisphosphate aldolase	gi|45330818 [*Antheraea yamama*i]	39.7/7.59	44/7.39	12	123	37%	-	-	-	**↑ **	**↑ **
50	large subunit arylphorin p76	gi|27802137 [*Heliothis virescens*]	30.6/6.33 (partial)	72/6.32	9	91	35%	-	-	-	**↑ **	**↑ **
52	60 S acidic ribosomal protein P0	gi|18253041 [*Spodoptera frugiperda*]	34.0/6.34	37/6.35	11	168	53%	-	-	-	**↑ **	**↑ **
55	calponin/transgelin	gi|108875705 [*Aedes aegypti*]	19.2/7.77	18/7.75	9	121	43%	1	61	KYGVPEEEIFQTADLFERR	**↑ **	**↑ **
58	eukaryotic translation initiation factor 4A	gi|95102876 [*Bombyx mori*]	48.0/5.1	53/5.26	29	238	53%	-	-	-	**No**	**↓ **
59	ATP synthase beta subunit	gi|115345328 [*Bombyx mori*]	54.9/5.32	54/5.05	25	236	55%	5	211	RIINVIGEPIDERG KAHGGYSVFAGVGERT RVALTGLTVAEYFRD KVSLVYGQMNEPPGARA RLVLEVAQHLGENTVRT	**No**	**↓ **
60	proteasome alpha 3 subunit	gi|114051245 [*Bombyx mori*]	28. 4/5.27	31/5.3	11	100	30%	2	67	KLYEPGANKRI KAVENSGTVIGLRG	**No**	**↓ **
61	heat shock protein 1	gi|56462154 [*Lonomia obliqua*]	21.9/6.32	27/5.75	7	90	27%	-	-	-	**No**	**↑ **

Symbols in the table: *, PMF score > 74 significant, MS/MS score > 45 significant; A, protein variation; P, phosphorylation variation; -, not known; ↑, increase compared to 5th-F; ↓ decrease compared to 5th-F; No, no change.

Most of the experimental MWs and pIs of the identified proteins are consistent with their theoretical values, but the MWs and pIs of a few proteins including spots 36, 37, 40, 44, 45 and 50 in the gel varied, presumably due to post-translational modification. Phosphorylation may increase the experimental MW and shift the protein to a more acidic pI, as for heat shock protein (HSP1) (spot 40, 61) and solute carrier family 25 (spot 45). Moreover, the MW difference between protein homologues should also be taken into consideration since the majority of proteins were identified based on protein sequence information from other species. For example, the theoretical MW of aldehyde dehydrogenase from *Bombyx mori *is 53 kDa which is different from the approximately 70 kDa experimental MW of aldehyde dehydrogenase in *H. armigera*. Nevertheless, further verification is needed for this protein.

In the identified proteins, hexamerin (spot 27, 28, 29) and HSP1 (spot 40, 61) appeared more than once. The spots of hexamerin were very close to each other in the gels, so they might result from different isoforms or other post-translational modifications. However, the MWs of the two HSP1s (spot 40 and spot 61) were different so it is possible that they are different HSPs.

### Analysis of identified proteins on the gene transcription level

According to a previous study on the developmental stages of *H. armigera *[13]*, Helicoverpa *larval metamorphosis begins about 72 h after larvae enter their last instar. Therefore, total RNA for RT-PCR analysis was extracted from the feeding 5th instar larvae (5th-F, 5th-24 h), the feeding 6th instar larvae (6th-F, 6th-24 h, 6th-48 h) and metamorphically committed larvae (6th-M, 6th-72 h, 6th-96 h, 6th-108 h).

**Figure 3:**
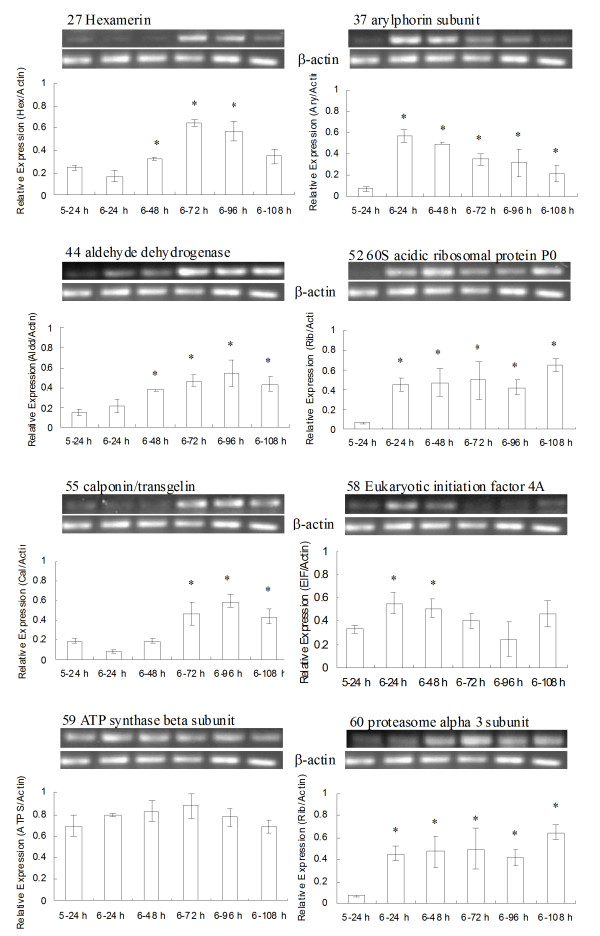
**Reverse Transcription PCR analysis of transcription of genes encoding proteins identified in 2D-electrophoresis**. Total RNA was isolated from 5th-feeding (5-24 h), 6th-feeding (6-24 h, 6-48 h), and 6th-metamorphically committed (6-72 h, 6-96 h, 6-108 h) larval epidermis. The β-actin gene was used for normalization of the compared templates. The gene expression ratio was calculated by Quantity One (Version 4.5, Bio-Rad, United States). All of the experiments were repeated at least three times. The values are mean ± standard deviation obtained by normalization of target genes against β-actin (*, difference is significant compared to 5-24 h by student *t *test, p < 0.05).

Eight genes encoding the identified proteins described above in Table 2 were analyzed. The results showed that hexamerin, calponin, 60 S acidic ribosomal protein P0, arylphorin subunit and aldehyde dehydrogenase appeared increased transcriptional expression during metamorphosis, which were consistent with the result of 2-DE (Figure 3).

**Table 2:** RT-PCR primers

Primer name	Forward primer	Reverse primer
Hexamerin	aggagcaacctctcgcagaaag	tgacgggaagacttcaggaag
Calponin	ccagactattgatttgtgggaga	cgttcttgtcagcctctttgg
Arylphorin subunit	aacaagattgagcgcaagtcc	ctaggcatgttgtcgggcac
60 S acidic ribosomal protein P0	caaggaggctaccaccatca	cccatgtcgtcgtcgctct
Aldehyde dehydrogenase	cgccctggtcaaggaagc	ggagccggtgaaagcaact
Eukaryotic translation initiation factor 4A	ctggcatcaggtcgtggaaa	tgcaaggcataatagcgcgt
ATP synthase beta subunit	gcccgtggtgtccagaag	gggcacgagccacagtcag
Proteasome 3 alpha subunit	tctgcgagggaaggatggt	catcggaaatgagacctgctact
Broad	atggctgatcaattctgttta	gttcggtgaagagaaattttc
β-actin	cctggtattgctgacggtatgc	ctgttggatggtggagagggaa

The eukaryotic translation initiation factor 4A (eIF 4A) showed an increase in transcription at the 6th-24 and 6th-48 h feeding stages but no significant variation after metamorphic commitment when larvae entered 6th-72 h. Similarly, the ATP synthase beta subunit did not show a significant increase during metamorphosis (Figure 3). These were consistent with the results of 2-DE, where they had the same expression levels between 5th-24 h and metamorphosis but with decreased phosphorylation levels during metamorphosis (Figure 2C). These two proteins might participate in metamorphosis by a regulated decrease in their phosphorylation levels but not in mRNA transcription levels.

The proteasome alpha 3 subunit transcription levels showed a typical increase in mRNA levels during metamorphosis, which was not correlated to the 2-DE result of constant protein level (Figure 3). The reason for this lack of correlation might be post-transcriptional regulation or increased protein turnover.

### Analysis of identified proteins on the translation level

**Figure 4:**
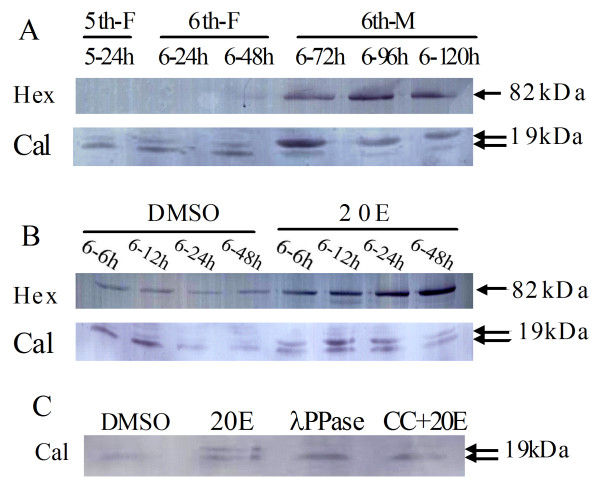
**Western Blot analysis of protein expressions in epidermis**. (A) Expression of hexamerin (82 kD) and calponin (19 kD) in the epidermis during larval development. 5th-F, fifth feeding larvae; 6th-F, sixth feeding larvae; 6th-M, 6th instar metamorphically committed larvae. 5-24 h, 6-24 h, 6-48 h, 6-72 h, 6-96 h and 6-120 h respectively denote samples from different developmental stages. (B) Effect of 20E injection on the expression of hexamerin and calponin in the epidermis. Same diluted DMSO was used as a control. 6-6 h, 6-12 h, 6-24 h and 6-48 h are the developmental ages of larvae. (C) Analysis of phosphorylation of calponin, same diluted DMSO as a control; 20E, sample from 6-12 h larvae injected with 20E; λPPase, sample from same 20E-injected larvae and treated with λPPase; CC+20E, sample from 6-12 h larvae injected with CC and 20E.

Western blotting was used to confirm expression levels of differentially expressed proteins identified by the proteomic approach. Because the antibodies against *H. armigera *proteins were not commercially available, only two identified proteins, calponin (Cal) and hexamerin (Hex) were adopted for analysis in the epidermis of the 5th-F, 6th-F and 6th-M larvae. Consistent with the 2-DE results, calponin and hexamerin both showed an increase at 6th-72 h, 6th-96 h and 6th-120 h during metamorphosis (Figure 4A). These results suggested that both genes were up-regulated by 20E *in vivo *during metamorphosis.

To test this hypothesis, the 6th instar feeding larvae were injected with 20E and protein samples were extracted. Western blot analysis showed that both calponin and hexamerin underwent a remarkable enhancement in their expression levels after 20E injection compared to the DMSO control. These results indicate that 20E up-regulated the expression of calponin and hexamerin during metamorphosis of the cotton bollworm (Figure 4B).

As shown in Fig. 4B, calponin exhibited two bands with slightly different molecular sizes when stimulated by 20E. Since *Helicoverpa *calponin does not have signal peptides and is unlikely to be exposed to the N-glycosylation machinery for glycosylation http://www.cbs.dtu.dk/services/NetNGlyc/, the upper band is most likely the phosphorylated form of calponin. To test this hypothesis, the protein sample was treated with λ phosphatase. λ phosphatase treatment decreased the upper band signal and enhanced the lower band signal, confirming that the upper band is a phosphorylated form of calponin (Figure 4C). Protein kinase consensus recognition sequence analysis revealed that calponin contains a Protein kinase C (PKC) phosphorylation site at amino acid residue Thr_183 _with score 0.78 http://www.cbs.dtu.dk/services/NetPhosK/. This prompted us to test whether PKC is involved in the phosphorylation of calponin. When the PKC specific inhibitor (chelerythrine chloride, CC) was injected into the larvae together with 20E, the upper band disappeared (Figure 4C) and the lower band immunoreactivity doubled, indicating that PKC is responsible for calponin phosphorylation.

### Investigating the pertinence of calponin to metamorphosis

**Figure 5:**
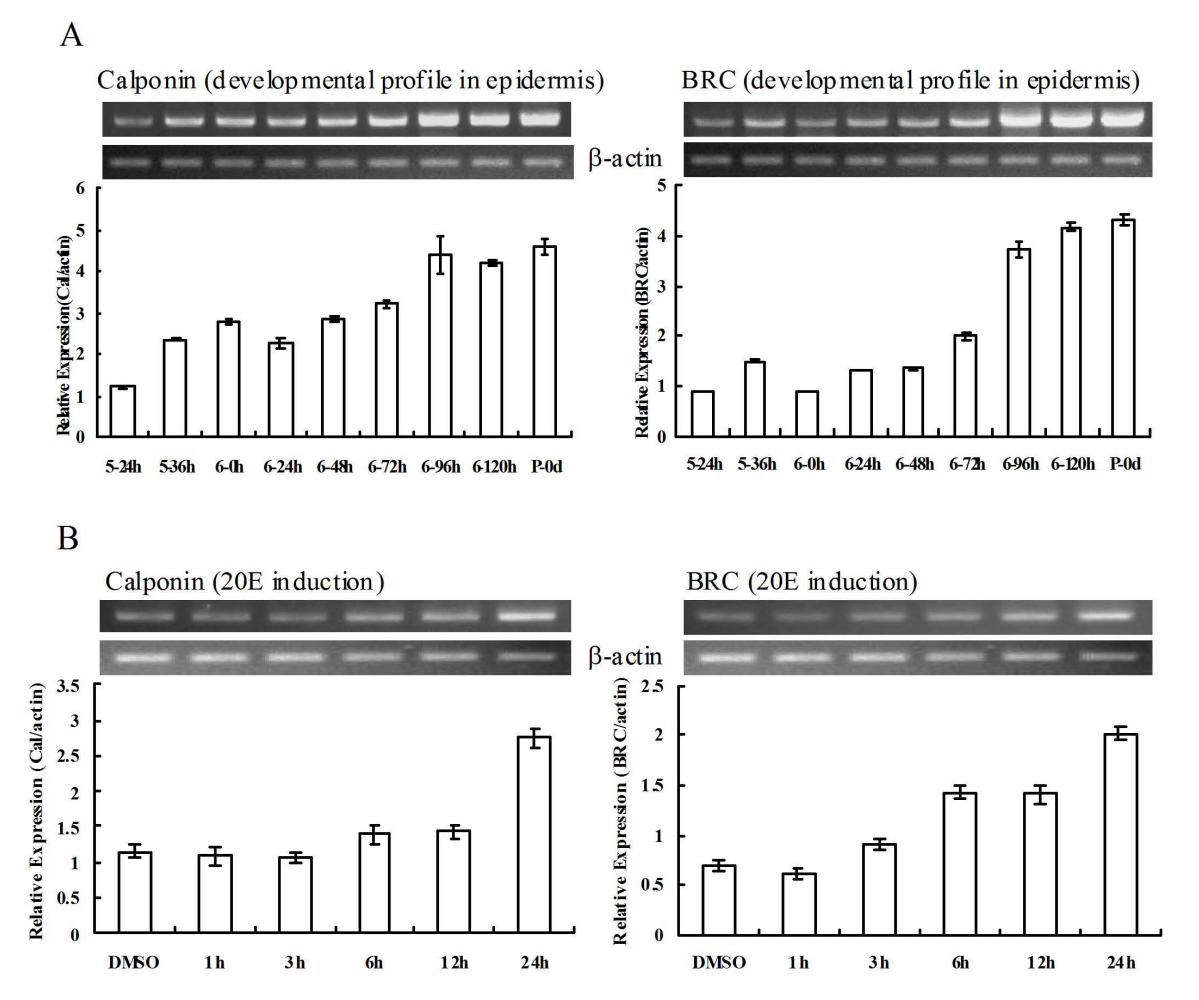
**Developmental profiles and hormone induction of calponin and Br in epidermis**. (A) Expression changes of both genes. 5-24 h, 5-36 h, 6-0 h, 6-24 h, 6-48 h, 6-72 h, 6-96 h, 6-120 h, P-0 day denote mRNA samples from different developmental stages from 5th-instar 24 hour to pupae. (B) 20E induction. 6th-6 h larvae were injected with 20E (500 ng/larva) for 1 h, 3 h, 6 h, 12 h and 24 h, DMSO injection as a control.

To examine the pertinence of calponin to metamorphosis, we examined the expression profiles of calponin and Broad (Br), an initiating factor of metamorphosis. Results showed that the mRNA of calponin in the epidermis increased when larvae entered metamorphosis from 6th-72 h to pupae, which was correlated to the expression profile of Br. In addition, both calponin and Br were able to be induced after 20E was injected into the 6th-6h larva for 24 hours (Figure 5).

## Discussion

Calponin is an actin-binding protein that inhibits actomyosin ATPase activity [14]. In human muscle, calponin can directly activate PKC autophosphorylation that increases PKC activity [15]. Since PKC is required for 20E-induced gene expression and can block JH action [3,6,16], we hypothesized that up-regulation of calponin during the larval-pupal metamorphosis may facilitate 20E action by activating PKC. Interestingly, calponin can also be phosphorylated by PKC [17], but its phosphorylation signal in the present study was weak in 2-DE gels during the larval-pupal metamorphosis. The low phosphorylation signal might be due to a single putative PKC phosphorylation site as revealed by the protein kinase consensus recognition sequence analysis described above. Nevertheless, we detected the phosphorylation of calponin by Western blot and demonstrated that 20E increases calponin phosphorylation via PKC.

The mRNA of calponin and Br in epidermis of *H. armigera *exhibited similar expression profiles. They both vastly accumulated during metamorphosis. Both calponin and Br can be induced by 20E when 6th feeding larvae were injected with 20E for different time periods. This induction seemed to be distinguishing. Induction of Br mRNA by 20E was earlier than that of calponin. These validated the story that Br initiates metamorphosis. Furthermore, calponin is demonstrated to be possibly involved in larval-pupal metamorphosis of *H. armigera*.

*Helicoverpa *calponin shares 70% identity with *Apis *Chd64 and 67% identity with *Drosophila *Chd64. Li et al. [18] presented a model of the signal transduction pathway for the JH-regulated genes, in which Chd64 is a chaperone protein that regulates gene expression in the 20E and JH signal transduction pathway. They indicated that in the presence of high JH and 20E levels (larval molting stage) the mRNAs of FKBP39 (39-kDa FK506-binding protein) and Chd64 (21-kDa calponin-like protein) are induced, interact with EcR (ecdysone receptor), USP and Met, and bind to the DmJHRE1 (*D. melanogaster *JH response element 1) sequence. This could result in the increase in the expression of DmJHRE1-containing genes and reduction in the expression of ecdysone response genes due to a decrease in the availability of EcR and USP. When 20E increases in the absence of JH (metamorphosis), the FKBP39 and Chd64 levels are low. Under this circumstance, EcR heterodimerizes with other members of the nuclear receptor superfamily, especially with USP, thus inducing the expression of ecdysone response genes. However, our results show that calponin was up regulated during metamorphosis and 20E increased the expression of calponin. This discrepancy may be due to the differences among species or the differences among proteins. Because Chd64 has been shown to interact with 16 proteins http://www.thebiogrid.org/SearchResults/summary/63970, the exact function of calponin in metamorphosis needs further study.

Hexamerins usually consist of six approximately 80 kDa subunits, giving rise to a native molecule of about 500 kDa [19]. Hexamerins containing a high concentration of aromatic amino acids are called arylphorins [20]. They are synthesized and reach extraordinary concentrations just before metamorphosis [19]. Although hexamerins are thought to act mainly as storage proteins, they have also been identified as constituents of the sclerotizing system [21], which plays a key role in pupal cuticle formation. Phylogenetic analysis revealed that insect prophenoloxidases were also members of hexamerin family, and played a role in sclerotization of the cuticle and encapsulation of foreign particles [22]. Zhou also hypothesized that hexamerins limited the impacts of JH on termite caste polyphenism by modulating JH availability [23]. By subtractive hybridization, Dong et al. pointed out that hexamerin was also up-regulated at the transcriptional level in the epidermis of *H. armigera *during metamorphosis [11].

A viral-like chitinase was also found to be up-regulated. The degradation of cuticular chitin by chitinases is a vital step prior to ecdysis and metamorphosis [24]. Kramer et al. [25] reported that the chitinase gene of *Manduca sexta *was most highly expressed in epidermal and gut tissues during the larval-pupal metamorphosis and its transcription was stimulated by 20E and inhibited by a JH mimic. The chitinase might be involved in remodeling of the integument during metamorphosis.

Proteasome alpha 3 subunit is a member of the 20S proteasome, which is ATP-independent and has several distinct catalytic activities involved in the ubiquitin-proteasome system [26,27]. Phosphorylation can control the import of proteasomes into the nucleus [28]. The proteasome subunit alpha type 5 was found up-regulated in fat bodies of *H. armigera *during larval molting [12]. The up-regulation of proteasome alpha 3 subunit suggested the participation of proteasomes in tissue remodeling during larval molting and metamorphosis.

Some of the differentially expressed proteins identified in the metamorphic larval epidermis in the present study, such as ATP synthase, proteasome subunit and translation initiation factor 4A, are similar to those found in the molting larval epidermis in our previous study [12]. Interestingly, ATP synthase beta subunit and eukaryotic translation initiation factor 4A, which were induced by 20E and inhibited by the PKC specific inhibitor chelerythrine chloride (CC) in *Drosophila *salivary glands [6], are also phosphoproteins in the metamorphosis epidermis in our study, suggesting that they might play important roles in the 20E signal transduction pathway during metamorphosis.

## Conclusion

The results suggest that the up-regulation and/or changes in the phosphorylation levels of proteins are involved in the larval-pupal metamorphosis. Hexamerin was highly expressed in the epidermis during metamorphosis. In addition, calponin was highly expressed in the metamorphic epidermis and phosphorylated by protein kinase C, which suggesting it plays an important role in metamorphosis.

## Methods

### Experimental animals

Larvae of the cotton bollworm (*H. armigera*) were reared on an artificial diet described (made from powder of wheat germs and soybeans with various vitamins as well as inorganic salts) by Zhao et al. [29] at 28°C with 60-70% relative humidity in our laboratory. The light:dark schedule was 14:10 h.

### Preparation of protein samples

For 2-DE analysis, we used samples from fifth-instar feeding larvae (5th-F) and sixth-instar metamorphically committed larvae (6th-M). The 5th-F larvae were defined as the larvae that had shed their old fourth instar cuticle and had grown for 24 h. The 6th-M larvae were defined as the larvae at 72 h, 84 h, 96 h, 108 h, and 120 h after molting into the 6th instar.

The epidermis was dissected from the 5th-F larvae or the 6th-M larvae at the indicated time points, homogenized by glass homogenizer in 1 ml sample buffer [40 mM Tris, 3 mM EDTA, 1 mM phenylmethanesulfonyl fluoride], and centrifuged at 12,000 rpm (24,300 g) for 10 min at 4°C. The supernatants were passed through 0.45 μm microporous filters (Xing Ya, Shanghai, China). Total proteins in the samples were determined by the Bradford method 30. The metamorphosis sample was a mixture of 1 mg protein sample from each of the five stages of the sixth instar larvae described above.

Each protein was precipitated with two volumes of acetone containing 10% (w/v) trichloroacetic acid and 20 mM dithiothreitol (DTT) at -20°C for 2 h. After being centrifuged at 12,000 rpm (24,300 g) for 15 min, the precipitate was washed twice with two volumes of pre-cooled acetone containing 20 mM DTT for 15 min and centrifuged to collect the proteins. The proteins were then dried at room temperature.d

### Two-dimensional gel electrophoresis (2-DE)

The dried protein samples were redissolved in a rehydration solution containing 8 M urea, 2 M thiourea, 4% 3-[(3-Cholamidopropyl)dimethylammonio]propanesulfonic acid, 65 mM DTT and 0.2% (w/v) Ampholyte pH 3.0-10.0 (GE Healthcare, United States). Eight hundred micrograms of protein were loaded on an 18-cm nonlinear Immobiline IPG Drystrip (pH 3-10) (GE Healthcare) in a rehydration tray for 14 h. An Ettan IPGphor3 system (GE Healthcare) was used for the first-dimensional isoelectric focusing (IEF) at 250 V for 1 h, 500 V for 1 h, 1,000 V for 5 h, followed by linearly ramping to 10,000 V over 3 h and then holding at 10,000 V until 60,000 V-h had been accumulated. After IEF, the IPG strip was first equilibrated with an equilibration buffer (0.375 M Tris-HCl, pH 8.8, 6 M urea, 20% glycerol, 2% SDS) containing 65 mM DTT for 10 min followed by 135 mM iodoacetamide for 10 min with constant shaking. After equilibration of the focused IPG strip, the strip was transferred to the top of a 12% SDS polyacrylamide gel and sealed with 1% low melting agarose. The second-dimensional electrophoresis was performed on a Protean II xi system (Bio-Rad) at 10 mA per gel for 30 min, and then 25 mA for approximately 6 h. Three independent repeats were performed. The detailed protocol for 2-DE is described in the instruction manual from GE Healthcare.

### Protein staining and imaging

After electrophoresis, the gels were first stained with the Pro-Q Diamond Phosphoprotein Stain (Molecular Probes, United States) following the manufacturer's instructions. Briefly, the gels were fixed with 50% (v/v) methanol and 10% (v/v) acetic acid for 60 min twice. After washed with ultrapure water, the gels were incubated in the dark in Pro-Q Diamond Phosphoprotein Stain with gentle agitation for 1.5-2 h. The gels were destained with a destaining solution [20% (v/v) acetonitrile, 50 mM sodium acetate, pH 4.0] 3 times, 30 min each. The gels were imaged with a Typhoon Trio^+ ^System (GE Healthcare, United States) and the images were optimized using phosphoprotein molecular weight standards (Invitrogen, United States). Then the gels were stained with colloidal Coomassie Brilliant Blue G-250 [10% (w/v) ammonium sulfate, 1% (w/w) phosphoric acid, 0.1% Coomassie Blue G-250] and destained with ultrapure water. The gel images were analyzed using ImageMaster 2D Platinum 6.0 software (GE Healthcare) to identify differentially expressed and phosphorylated proteins.

### In-gel digestion

The differentially expressed and phosphorylated protein spots were manually picked from the gel and placed individually into methanol-treated tubes. Each gel piece was washed 3 times with distilled water. Then 200 μL of 200 mM ammonium bicarbonate with 40% acetonitrile was added to each tube and incubated at 37°C for 30 minutes. After that, the solution was removed from the tube. The gel piece in each tube was suspended in 100 μL acetonitrile and dehydrated for 5 min before the excess acetonitrile was discarded. The gel piece was dried in vacuum for 15 min and then treated with 5 μL of a trypsin solution [20 μg/mL trypsin (Proteomics Grade, Sigma, United States), 40 mM ammonium bicarbonate, 9% acetonitrile]. The tube was incubated on ice for 45 min and then excess trypsin solution was removed. To cover the gel piece, 5 μL of 40 mM ammonium bicarbonate in a 9% acetonitrile solution was added and incubated overnight at 37°C. After the incubation, the liquid from the gel piece was transferred to a new labeled tube. Then 5 μL of 0.1% trifluoroacetic acid in a 50% acetonitrile solution was added to the gel piece and incubated for 30 min at 37°C. After that, the solution was collected and combined with the liquid from the previous step. The combined sample solution was used for MALDI-TOF-MS (matrix assisted laser desorption/ionization time-of-flight mass spectrometry) analysis.

### MALDI-TOF-MS and MS/MS analysis

The trypsin-digested peptides were mixed with a MALDI matrix [7 mg/mL α-cyano-4-hydroxycinnamic acid, 0.1% trifluoroacetic acid and 50% acetonitrile] and spotted on the MALDI target plates. MS and MS/MS spectra were obtained with an ABI 4700 MALDI-TOF/TOF mass spectrometer (Applied Biosystems, United States) operating in a result-dependent acquisition mode. Peptide mass maps were acquired in a reflectron mode (1000 V accelerating voltage) with 1,000 laser shots per spectrum. Six external standards (mass standard kit for the 4700 Proteomics Analyzer calibration mixture, Part Number 4333604, Applied Biosystems) were used to calibrate each spectrum to a mass accuracy within 50 ppm. The MS/MS data were acquired with stop conditions and 3,000-6,000 laser shots were accumulated for each spectrum (Additional files 1). MS/MS analysis were performed at collision energy of 1 kV and the collision gas pressure of 2.0 × 10^-7 ^to 3 × 10^-8 ^Torr.

The MASCOT search engine (version 1.9, Matrix Science, http://www.matrixscience.com/search_form_select.html) was used to search all of the tandem mass spectra against NCBInr_Metazoa Database. Carbamidomethyl (C) (cysteine carbamidomethylation) and Oxidation (M) (methionine oxidation) were selected as fixed and variable modifications. One missing trypsin cleavage was allowed. Peptide mass tolerance and fragment mass tolerance were set to 100 ppm or 0.6 Da. High confidence identifications had statistically significant search scores (greater than 95% confidence, equivalent to MASCOT expect value p < 0.05), were consistent with the protein's experimental pI and MW, and accounted for the majority of ions present in the mass spectra.

### Reverse Transcription PCR

Total RNA was isolated from the epidermis of the 5th-F, 6th-F and 6th-M larvae. In 20E-induction experiment, total RNA was extracted from epidermis of larvae injected with 20E for different time periods. Five micrograms of RNA was used to reverse transcribe the first strand cDNA (First Strand cDNA Synthesis Kit, Sangon, China), which was then used as a template (0.5 ng) in PCR reactions with gene-specific primers. Specific PCR primers for the genes of the proteins identified in MALDI-TOF-MS were designed from expression sequence tags of *H. armigera *(Table 2). PCR cycles were as follows: one cycle (94°C, 2 min); 27 cycles (94°C, 30 s; 55°C, 40 s; 72°C, 30 s), followed by a last cycle (72°C, 10 min). A β-actin gene fragment from *H. armigera *was also amplified as a control. Each RT-PCR assay was replicated three times.

### Western blotting

To confirm expression changes for some of the interesting proteins identified, the epidermis of fifth feeding larvae (5th-F), sixth feeding larvae (6th-F) and sixth-metamorphically committed larvae (6th-M) were dissected and homogenized for Western blotting. The protein extracted from each tissue was quantified by the Bradford method. Equal amounts of protein (50 μg) were subjected to 12.5% sodium dodecyl sulfate-polyacrylamide gel (SDS-PAGE) and then electro-transferred onto nitrocellulose membranes. The membranes were incubated in a blocking buffer (10 mM Tris-buffered saline) containing 2% milk power at room temperature for 1 h, then incubated with primary antibodies: diluted 1:100 for anti-calponin polyclonal antibodies (generated in our lab) and 1:500 for anti-hexamerin monoclonal antibody (a gift from Dr. Gang Ma, University of Adelaide, Australia) at 4°C overnight, respectively. Goat anti-rabbit IgG conjugated with horseradish peroxidase (HRP) diluted 1:10000 was adopted as a secondary antibody. 4-chloro-1-naphthol was used as a HRP substrate for visualizing the peroxidase activity. The quantity of protein loaded was controlled by SDS-PAGE with two gels simultaneously, one for transferring and the other for Coomassie Brilliant Blue staining.

Induction with 20E was performed as follows. A 10 mg/mL stock solution of 20E in dimethyl sulfoxide (DMSO) was diluted 1:100 in PBS (140 mM NaCl, 2.7 mM KCl, 10 mM Na_2_HPO_4 _and 1.8 mM KH_2_PO_4_). The 6th instar feeding larvae (6th-6 h, 6th-12 h, 6th-24 h and 6th-48 h) were each injected with 500 ng 20E in 5 μl of the dilution, and then incubated for 12 h. An equal volume of DMSO diluted in PBS was injected into control larvae.

To verify the identified phosphoprotein, λ protein phosphatase (λPPase) was added to the epidermis homogenate of 20E-injected larvae at a final concentration of 4 μg/100 μl reaction buffer [500 mM Hepes (N-2-hydroxyethylpiperazine-N-ethane-sulphonicacid), pH 7.5, 1 mM EDTA, and 20 mM MnCl_2_] and incubated at 37°C for 10 min before subjected to Western blot analysis. To reveal whether phosphorylation of the identified protein was regulated by protein kinase C (PKC), a PKC specific inhibitor, CC (chelerythrine chloride), was injected into the larvae together with 20E. The epidermis proteins of each sample were extracted 12 h after injection and subjected to SDS-PAGE and Western blotting.

## Authors' contributions

QF and PCL performed experiments. JXW and QSS participated in the design and coordination of the work. XFZ conceived the study and helped to draft the final version of this manuscript. All authors read and approved the final manuscript.

## Supplementary Material

Additional file 1 **MS/MS spectrum**. The files contain the MS/MS spectrum information for 7 protein spots identified by tandem mass spectrum, which can reflect the m/z information. The files also contain a txt file which includes a corresponding relationship between the number of each PDF file and the gene name listed in Table 1. Click here for file
